# Integrating transformer and imbalanced multi-label learning to identify antimicrobial peptides and their functional activities

**DOI:** 10.1093/bioinformatics/btac711

**Published:** 2022-11-03

**Authors:** Yuxuan Pang, Lantian Yao, Jingyi Xu, Zhuo Wang, Tzong-Yi Lee

**Affiliations:** School of Science and Engineering, The Chinese University of Hong Kong, Shenzhen, Shenzhen 518172, China; Warshel Institute for Computational Biology, The Chinese University of Hong Kong, Shenzhen, Shenzhen 518172, China; School of Science and Engineering, The Chinese University of Hong Kong, Shenzhen, Shenzhen 518172, China; Warshel Institute for Computational Biology, The Chinese University of Hong Kong, Shenzhen, Shenzhen 518172, China; School of Life and Health Sciences, School of Medicine, The Chinese University of Hong Kong, Shenzhen, Shenzhen 518172, China; Warshel Institute for Computational Biology, The Chinese University of Hong Kong, Shenzhen, Shenzhen 518172, China; Warshel Institute for Computational Biology, The Chinese University of Hong Kong, Shenzhen, Shenzhen 518172, China; School of Life and Health Sciences, School of Medicine, The Chinese University of Hong Kong, Shenzhen, Shenzhen 518172, China

## Abstract

**Motivation:**

Antimicrobial peptides (AMPs) have the potential to inhibit multiple types of pathogens and to heal infections. Computational strategies can assist in characterizing novel AMPs from proteome or collections of synthetic sequences and discovering their functional abilities toward different microbial targets without intensive labor.

**Results:**

Here, we present a deep learning-based method for computer-aided novel AMP discovery that utilizes the transformer neural network architecture with knowledge from natural language processing to extract peptide sequence information. We implemented the method for two AMP-related tasks: the first is to discriminate AMPs from other peptides, and the second task is identifying AMPs functional activities related to seven different targets (gram-negative bacteria, gram-positive bacteria, fungi, viruses, cancer cells, parasites and mammalian cell inhibition), which is a multi-label problem. In addition, asymmetric loss was adopted to resolve the intrinsic imbalance of dataset, particularly for the multi-label scenarios. The evaluation showed that our proposed scheme achieves the best performance for the first task (96.85% balanced accuracy) and has a more unbiased prediction for the second task (79.83% balanced accuracy averaged across all functional activities) when compared with that of strategies without imbalanced learning or deep learning.

**Availability and implementation:**

The source code and data of this study are available at https://github.com/BiOmicsLab/TransImbAMP.

**Supplementary information:**

Supplementary data are available at *Bioinformatics* online.

## 1 Introduction

Currently, there is a decrease in the number of effective antimicrobial agents, referred to as the post-antibiotic era (Kwon and Powderly, 2021). Many known pathogens persist owing to rapid variation to escape existing drug treatments, which disarms therapies and leads to severe patient outcomes (Chandra *et al.*, 2021). Novel therapeutic options have emerged as alternatives to traditional treatments. Antimicrobial peptides (AMPs) are short amino acid sequences originating from organisms and are promising candidates for dealing with antimicrobial resistance (AMR) (Rima *et al.*, 2021). AMPs have been reported to be potential treatment options for bacterial or viral infections and carcinoma (Pen *et al.*, 2020; Roudi *et al.*, 2017; Shao *et al.*, 2018). AMPs are able to penetrate membranes and interact with membrane proteins of the target microbes (Mahlapuu *et al.*, 2016). These modes of action enable AMPs to have specific advantages, such as broad-spectrum inhibition activities and persistent effectiveness against pathogens exhibiting AMR.

The discovery of novel AMPs has promoted the development and maintenance of AMP analysis platforms and databases. AMPs repositories provide abundant information, enabling researchers to analyze their mechanisms of action. For example, the latest version of dbAMP (Jhong *et al.*, 2021) collected 26 447 AMPs and 2262 antimicrobial proteins from 3044 organisms. DRAMP (Shi *et al.*, 2021) contains 22 259 entries, of which over 2000 new entries have been recently added. LAMP (Ye *et al.*, 2020) provides cross-links with other AMP databases as proxies to access different characterized properties. Additionally, some repositories also provide annotations of activities toward specific microorganisms. For example, AVPdb (Qureshi *et al.*, 2013) has collected over 2683 antiviral peptides targeting ∼60 common viruses. CancerPPD (Tyagi *et al.*, 2015) curates the sequence data of anticancer peptides and proteins. The Hemolytik database (Gautam *et al.*, 2014) describes experimentally verified hemolytic and non-hemolytic peptides for recording toxicity in mammals. Computer-aided methods, especially those with machine learning-based strategies, provide fast and efficient proteome and synthesis sequence screening alternatives to accelerate the discovery of novel AMPs. For example, ClassAMP (Joseph *et al.*, 2012) was developed combining Random Forest (RF) and Support Vector Machine (SVM) with composition and physiochemical peptide descriptors to predict the antibacterial, antiviral, and antifungal activities of AMPs. Similarly, iAMPpred (Meher *et al.*, 2017) uses an SVM by incorporating structural features to improve the prediction performance. In addition to the machine learning methods, deep learning methods have also been used to identify AMPs. The formation of natural proteins or peptides can be analogous to natural language, which enables deep learning to directly and accurately decipher information within peptide sequences (Yang *et al.*, 2019). In Veltri *et al.* (2018), a deep learning model that integrates convolution and long short-term memory layers were built to identify potential AMP sequences. Bidirectional encoder representation from transformers (BERT) (Devlin *et al.*, 2019) has achieved significant success in many natural language processing (NLP) tasks. Zhang *et al.* (2021b) proposed a novel AMP recognition algorithm based on BERT, which improved the accuracy of AMP prediction.

Class imbalance, depicted as an unequal or distorted distribution across known classes within the dataset under classification problems, exists widely within the real-world implementation of machine learning (Lin and Xu, 2016). Various over- and under-sampling methods have been proposed to relieve the curse of class imbalance (Dong and Wang, 2011; Luengo *et al.*, 2011). The synthetic minority over-sampling technique (SMOTE) (Chawla, 2009; Chawla *et al.*, 2002) is one of the most effective methods and has been used in bioinformatics research (Xiao *et al.*, 2015). Moreover, focal loss (Lin *et al.*, 2017) was used to overcome class imbalance under deep learning objective detection problems and achieved significant success.

In this study, a novel deep learning-based method, TransImbAMP, is proposed for computer-aided AMP prediction. Two tasks are considered for the implementation of the model: the binary classification of identifying AMPs and predicting the functional activities of AMPs toward different targets in a multi-label manner. The method utilizes a transformer architecture with transferred knowledge from a large amino acid sequence collection to better encode peptide information. This method also adopts an imbalanced learning strategy with a modified loss function to relieve severe class imbalance. The results revealed that transfer learning and imbalanced learning could be integrated to make reliable improvements to biological sequence prediction.

## 2 Materials and methods

### 2.1 Dataset preparation

AMPs with validated sequence records and annotations of their targets were collected from several general AMP databases, including dbAMP (Jhong *et al.*, 2021), DRAMP (Shi *et al.*, 2021) and DBAASP (Pirtskhalava *et al.*, 2020). Seven functional activities toward different targets were selected as the following labels: gram-positive bacteria, gram-negative bacteria, viruses, fungi, cancer cells, mammalian cell inhibition, and parasites. Additionally, more sequences were extracted from databases [AVPdb (Qureshi *et al.*, 2013), AntiCP (Agrawal *et al.*, 2020) and AntiFP (Agrawal *et al.*, 2018)] with specific target domains to reinforce the quantity of the dataset, including annotated activities toward specific microorganisms. The replicated entries were removed by independently performing CD-HIT (Li and Godzik, 2006) with a 100% threshold on sequences under each target domain. The peptides without antimicrobial functions (non-AMP) were collected from UniProt (UniProt Consortium, 2020), with entries excluded using the following key words: ‘membrane’, ‘toxic’, ‘secretory’, ‘defensive’, ‘antibiotic’, ‘anticancer’, ‘antiviral’ and ‘antifungal’. The imbalance between AMP and non-AMP data was alleviated by reducing the size of non-AMP sequence entries using CD-HIT with a 40% threshold. The resulting dataset consisted of 6460 AMP and 15 921 non-AMP sequences.

In this study, we investigated two AMP prediction tasks. The first is to identify AMPs from broad-spectrum amino sequences, which is considered a common single-label binary classification task (AMP versus non-AMP). The second task predicts the functional activities of AMPs toward seven different targets, including gram-positive bacteria, gram-negative bacteria, fungi, viruses, cancer cells, parasites and function of mammalian inhibition. Two or more functional activities may exist for the same AMP sequence; therefore, this situation is identified as a multi-label classification problem (Tarekegn *et al.*, 2021). To pursue a fairer evaluation, we adopted a stratified method (Sechidis *et al.*, 2011) to split the dataset into a proper ratio, especially for multi-label functional activities. The size of the dataset for training and testing is presented in Supplementary Table S1.

### 2.2 Self-supervised model based on transformer to predict AMPs and their functional targets

A BERT architecture (Devlin *et al.*, 2019) was adopted to extract the sequence information of the input sequences. The transformer uses a self-attention mechanism (Vaswani *et al.*, 2017) to automatically capture the intrinsic relationships between all possible amino acid pairs within the input sequence to improve the representation. The *L*-length input amino acid sequence is represented as $\left(s_{1},s_{2},\ldots ,s_{L}\right)$, where the *s_i_* are the tokens of the *i*-th amino acid (i=1,…,L) for the given sequence. The attention scores $a(s_{i},s_{j})>0$ are computed with respect to every pair of terms within a sequence. The input of the self-attention block is denoted as *V*, and the corresponding output as *A*. Computation is based on a scaled dot-product operation, which is expressed as:

(1) $$C=\text{softmax}(\frac{(VW_{1}){\left(VW_{2}\right)}^{T}}{\sqrt{d}})$$

(2) $$A=C^{T}(VW_{3}),$$

where *d* is specified as the dimension of the self-attention head. *W*_1_, *W*_2_ and *W*_3_ are learnable parameter matrices for transforming the input into its corresponding query, key and value forms, respectively. The transformer also utilizes multi-head self-attention with concatenated heads to further exploit the information between sequences. Several such layers can be stacked during end-to-end training to obtain a deep context-aware representation.

The pretrained BERT model from the TAPE archive (Rao *et al.*, 2019) was used as the backbone for TransImbAMP. The pre-training procedure was performed using the Pfam dataset (Mistry *et al.*, 2020), including over 31 million amino acid sequences, in a self-supervised fashion by masked-token prediction that predicts the masked token based on the context provided by the other unmasked token within the given sequence, i.e. calculating $p(s_{\text{masked}}|s_{\text{unmasked}})$. During pre-training, 15% of the input tokens were masked randomly. Consequently, the model utilizes the unlabeled sequence data through a pre-training procedure to obtain a relatively complete knowledge of the amino acid sequences and transfers this to downstream tasks to improve prediction performance.

**Fig. 1.**
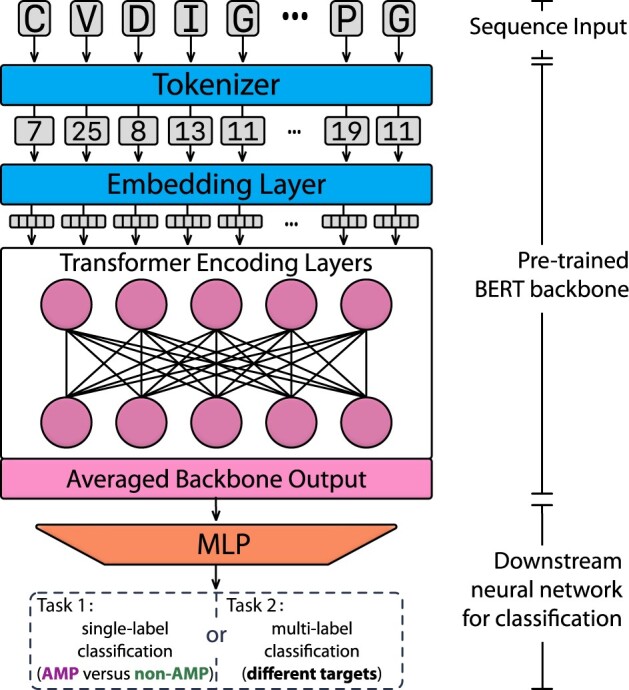
Architecture of the AMP prediction model using self-supervised transformer. The Tokenizer inputs the sequence and maps the amino acids to their corresponding numerical tokens. The embedding layer with learnable weights then receives the tokens, converts each of them to a 768-dimension vector, and feeds them into the transformer encoding layers. The output of the transformer encodings is averaged along with tokens and sent into the downstream neural network composed by a multi-layer perceptron (MLP). The number of the output neurons is decided by the prediction tasks

The backbone architecture is composed of 12 hidden layers, each of which consisted of 12 self-attention heads with 64 dimensions. Therefore, the output of the backbone for each token is a 768-dimension (referred to as the size of each hidden layer) representation in the last layer. To complete the classification tasks, the backbone outputs are averaged across the token dimension and fed into a two-layer neural network (NN) for downstream prediction. In the downstream NN, 640 hidden units with LeakyReLU (Maas *et al.*, 2013) activation are utilized, and Dropout (Srivastava *et al.*, 2014) is included to improve the generalization performance. During the fine-tuning (He *et al.*, 2019) process, the transformer backbone was frozen, and parameters of the downstream NN were updated iteratively to fit the classification tasks. The number of neurons for the final output layer differs for each task: two for the binary classification and seven (corresponding to the number of different functional activities) for the multi-label classification of AMPs’ targets. The architecture of the model is presented in Figure 1.

### 2.3 Multi-label classification with asymmetric loss

The dataset contains an imbalance between positive and negative labels, especially within the multi-label classification. Supplementary Table S1 shows the ratio of positive labels under different tasks for the dataset, indicating that an imbalance exists throughout both tasks. Class imbalance is the most common problem in multi-label classification because samples may contain many negative labels but few positive labels. To resolve the issue of imbalance and improve the predictive performance, an asymmetric loss (ASL) strategy (Ridnik *et al.*, 2021) is adopted for classification instead of traditional cross-entropy. The label of classification is denoted as *y*, and the output probability is represented as *p*. The ASL for the class-imbalanced prediction problem is defined as follow.

(3) $$L=\{\begin{array}{c} \begin{array}{cc} -{\left(1-p\right)}^{\gamma_{+}}\;\text{log}(p), & y=1\\ -p_{t}^{\gamma_{-}}\;\text{log}(1-p_{t}), & y=0, \end{array} \end{array}$$

where $\gamma_{+}$ and $\gamma_{-}$ are the focusing parameters with respect to the positive and negative classes. Specifically, under the condition of $\gamma_{+}=\gamma_{-}=0$, the above terms turn into a standard cross-entropy loss. $p_{t}=\max(p-t,0)$ is called the shifted probability with t≥0 as the probability margin. The ASL improves the traditional cross-entropy loss in two aspects. The first aspect is *asymmetric focusing*. By modifying the $\gamma_{+}\geq 0$ or $\gamma_{-}\geq 0$, the contribution of easily classified positive (p≫0.5) or negative (p≪0.5) can be down-weighted. Therefore, the model can focus on samples which are harder to be classified during the training process. In common classification scenarios, such as the AMP classification tasks in this study, the focusing parameters are set as $\gamma_{-}\geq \gamma_{+}$ because the positive class is often needed to emphasize and is deficient in the number of samples. The other aspect is the *probability shifting*. While the previous strategy attenuates the contribution of low-probability negative samples to loss (soft-thresholding), this mechanism introduces an additional ’hard-thresholding’ to improve the class imbalance. The loss of easy negatives and suspected mislabeled data with *p *<* t* is discarded as zero; therefore, their contribution is removed, which allows us to focus more on resolving samples that are difficult to classify. The final loss for multi-label classification is aggregated across all output logits corresponding to the labels.

### 2.4 Performance assessment and experimental settlement

Under the circumstances of the minority class, some evaluation indices are ambiguous and unfair (Briggs and Zaretzki, 2008). Therefore, metrics including sensitivity (SEN), specificity (SPEC), balanced accuracy (BA) and geometric mean (GMean) were selected, considering that there are varying extents of data imbalance. Denote TP, TN, FP and FN as the number of true positives, true negatives, false positives, and false negatives, respectively, the mentioned metrics in this study are defined as:

(4) $$\text{SEN}=\frac{\text{TP}}{\text{TP}+\text{FN}}$$

(5) $$\text{SPEC}=\frac{\text{TN}}{\text{TN}+\text{FP}}$$

(6) $$\text{BA}=\frac{\text{SEN}}{2}+\frac{\text{SPEC}}{2}$$

(7) $$\text{GMean}=\sqrt{\text{SEN}\times \text{SPEC}}.$$

The SEN and SPEC can present the rate of correctly classified instances of total positives or negatives. The BA and GMean (Bekkar *et al.*, 2013; Chou, 2001) are the arithmetic or geometric means for the SEN and SPEC, respectively, providing the balanced accuracies of the entire assessed dataset and have been widely adopted in previous research (Brodersen *et al.*, 2011; Zhang *et al.*, 2022).

Model fitting with acceptable predictive performance was ensured by training the model with 240 epochs for binary AMP classification and 300 epochs for multi-label target identification. The initial learning rate was set to 0.04 and the Adam optimizer (Kingma and Ba, 2015) was used for model fitting. Additionally, a step learning rate decay strategy was adopted to ensure better convergence. The learning rate decayed at the tipping points with different decay rates for both tasks. We assayed different experimental settings for the ASL hyperparameters, $\gamma_{+}$ and $\gamma_{-}$, to determine the best combinations based on the classification performance on the test dataset. Supplementary Table S2 summarizes the training settlement details. The TransImbAMP pipeline was established using the Pytorch package (Paszke *et al.*, 2019). The training process was applied using 4 × Nvidia 2080 Ti GPUs.

The machine learning-based method with sequence features was also applied as the baseline classifier for comparison with the deep learning-based methods. The features were designed similar to Pang *et al.* (2021), including amino acid composition (AAC), dipeptide composition (DPC), composition of k-spaced amino acid pairs (CKSAAGP), pseudo-amino acid composition (PAAC) and eight different physicochemical features. We selected RF (Breiman, 2001) as the baseline classifier owing to its comparative performance introduced by the nonlinear characteristics. The features utilized within the baseline method are described in the Supplementary Information.

## 3 Results

**Fig. 2.**
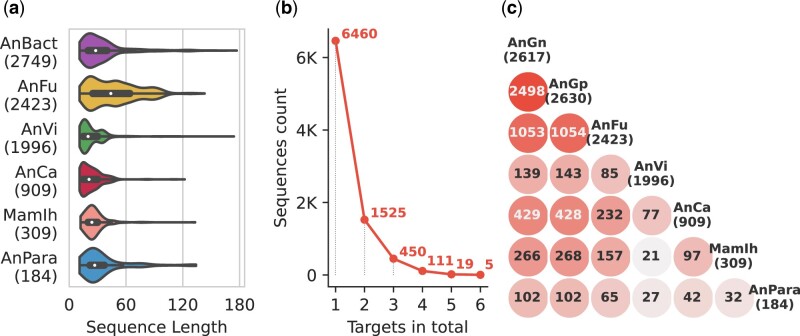
Statistics of the AMP collection. (**a**) The length distributions of AMPs categorized by their functional activity targets in the second task. (**b**) The number of peptide sequences according to their total surveyed targets. (**c**) The cross counted matrix for dual-functional sequences. Each off-diagonal element represents the number of peptide sequences that simultaneously possess two functional activities according to their diagonal target labels

The sequence lengths varied among the collected AMP and non-AMP sequences (Supplementary Fig. S1), with AMPs presenting shorter sequence length compared to that of peptides without antimicrobial activities. This coincides with the standpoint that AMPs maintain their membrane-interaction activities by shorter positively charged amino acid chains (Zhang *et al.*, 2021a). The length distributions categorized by the functional activities of different targets are presented in Figure 2a. Sequences with antifungal activities tend to be longer, whereas most antiviral, anticancer and mammalian cell inhibition sequences exhibited shorter sequences.

AMP-target prediction was delineated as a multi-label classification problem enabling the simultaneous identification of the functional activities of the queried AMPs for seven different targets. The number of sequences according to their functionally active targets are presented in Figure 2b. All antibacterial sequences were merged without considering the gram stain taxonomy. Over 2000 sequences exhibited multiple functional activities. Notably, five peptides preserved the activities of all investigated targets (Supplementary Table S3). Three of these peptides are from the cathelicidin family, which has been proven to be a broad-spectrum therapeutic agent with direct antimicrobial and immune modulation effects (Kościuczuk *et al.*, 2012). In addition, the number of dual-function sequences between each pair of functional activity targets is presented in Figure 2c. Major overlaps were observed within the dataset of gram-positive and -negative targeting functions. The antiviral category exhibited less concurrence with other activities, which may be related to the uniqueness of viral structures and modes of action toward viral infections (Kościuczuk *et al.*, 2012). These findings support the adoption of multi-label classification method to resolve AMP-target identification problems.

### 3.1 First task: AMP versus non-AMP

The transformer-based asymmetrical learning scheme was utilized to adapt the task of standard binary classification between AMP and non-AMP sequences. To substantiate the effect of asymmetric multi-label loss, experiments were performed by varying $\gamma_{+}$ and $\gamma_{-}$ from 0 to 5 and $\gamma_{-}\geq \gamma_{+}$ are simultaneously substantiated owing to the sample bias toward the negative data (non-AMP). In addition, a baseline model with RF was established to compare it with our proposed method. The concise performance, including the baseline classifier, transformer with cross-entropy, and transformer with ASL on the independent test dataset, are presented in Table 1. The benefit of the transferred knowledge from extensive amino acid sequence collection and language modeling architecture allowed the transformer model trained with cross-entropy loss to exceed the baseline method with 96.70% balanced accuracy and 96.69% GMean. Adopting the ASL (under the focusing parameters with $\gamma_{+}=1,\;\gamma_{-}=3$) led to the performance of TransImbAMP improving to 96.85% BA and 96.85% GMean. This can be seen from the slightly increased sensitivity (96.28%) and decreased specificity (97.43%) leading to more balanced results. The ASL relieves the sample bias between the positive and negative data. The test performance of the transformer-based models with 16 different combinations of the focusing parameters are presented in Supplementary Table S4. The focusing parameters of ASL allow us to control the classification results to concentrate on positives or negatives to different extents. A comparative performance analysis was conducted with other tools, including machine learning (Joseph *et al.*, 2012; Meher *et al.*, 2017) and deep learning-based (Veltri *et al.*, 2018) methods (Supplementary Table S5). These results demonstrate the superior performance of TransImbAMP with greater unbiased prediction of AMP identification. Consequently, the best performance was achieved with an appropriate proportion between $\gamma_{-}$ and $\gamma_{+}$.

**Table 1.** Evaluation performances on the test dataset of the first task: AMPs versus non-AMP

Method	BA (%)	SEN (%)	SPEC (%)	GMean (%)
Baseline	93.26	88.26	**98.26**	93.12
Transformer + cross-entropy	96.70	95.59	97.80	96.69
Transformer + ASL ($\gamma_{-}=3,\;\gamma_{+}=1$)	**96.85**	**96.28**	97.43	**96.85**

The best performance under each metric is highlighted in boldface.

### 3.2 Second task: functional activity identification of AMP targets

The second task of TransImbAMP is to identify the targets of AMPs, for which the model simultaneously predicts the functional activity of AMP toward gram-positive bacteria (AnGp), gram-negative bacteria (AnGn), fungi (AnFu), viruses (AnVi), cancer cells (AnCa), parasites (AnPara) and mammalian cell inhibition (MamIh). Therefore, the model was established with ASL to relieve the severe imbalance within the defined multi-label problem. We constructed a RF classifier for each target label as the baseline model and conducted a similar experiment for the focusing parameters. The best performance was 79.83% balanced accuracy, 78.24% GMean and 73.93% sensitivity aggregated across all seven labels using TransImbAMP with the ASL parameters of $\gamma_{-}=5,\gamma_{+}=1$ (Table 2). The transformer with cross-entropy or baseline classifier were inclined to predict negative labels with 56.82% or 49.88% sensitivity averaged across all labels. A similar decrease also occurred in balanced accuracy and GMean, which suggests that the impact of the sample class-imbalance issue was reduced by using ASL. The performances specific to different target labels are shown in Supplementary Table S6. The transformer with ASL achieved the best balanced accuracy and GMean for all targets, except fungi. Nonetheless, this guarantees the highest sensitivity for each target label. The baseline classifier and transformer with cross-entropy failed with placing excess emphasis on the negatives, as shown by the higher specificity. We also performed a comparative analysis with other available tools for microbial target prediction of AMPs (Supplementary Table S5). The results show the competitive performance of TransImbAMP with more balanced prediction results. Summaries of the average and comprehensive performances for different prediction targets are presented in Supplementary Tables S7 and S8, respectively. The results further illustrate the ability of ASL to downplay the contribution of easily classified negative samples and emphasize the contribution of positives.

**Table 2.** Performance metrics averaged across all the corresponding target label metrics under the second task

Method	BA (%)	SEN (%)	SPEC (%)	GMean (%)
Baseline	72.63	49.88	**95.37**	64.76
Transformer + cross-entropy	74.41	56.82	92.01	68.65
Transformer + ASL ($\gamma_{-}=5,\;\gamma_{+}=1$)	**79.83**	**73.93**	85.73	**78.24**

The best performance under each metric is highlighted in boldface.

### 3.3 Analysis of the encodings for investigated peptides

**Fig. 3.**
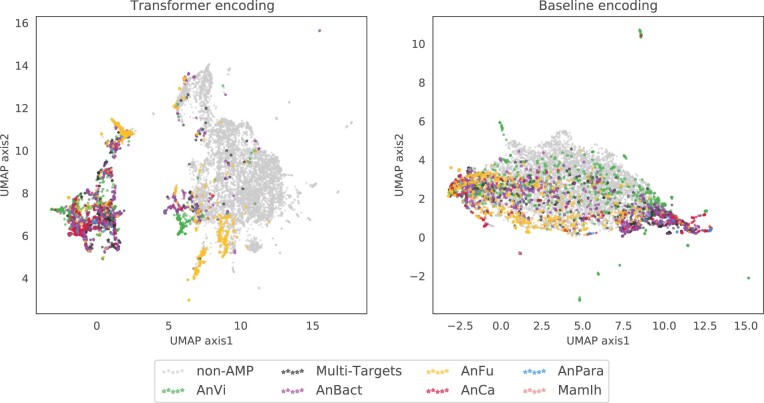
UMAP visualization of sequences for the proposed model (left) and the baseline (right) encoding

The transformer-based methods exhibited considerably improved performance compared with the baseline classifiers for both tasks. The primary reason for these improvements can be ascribed to the encodings of the transformer architecture. Therefore, we extracted feature encodings from the transformer backbone under the test dataset for non-AMP peptides and AMPs labeled with functional activities. A clear view of the feature representation was presented by adopting dimension reduction based on the uniform manifold approximation projection (UMAP) (McInnes *et al.*, 2018) to visualize the encodings by comparing them with the ordinary composition and physiochemical peptide descriptors from the baseline classifiers (Fig. 3). Transformer encodings exhibited significant effects on the intrinsic separation between AMP and non-AMP in the UMAP representation, while AMPs with different functional targets are also distinctly distributed. In contrast with the transformer, feature encodings of baseline methods cannot achieve the divergence of UMAP representation, for which many peptides with different functional abilities are entangled with others. This analysis demonstrates the capability of transformer-based models with transfer learning to generate more accurate representations for discriminating different functional peptides.

## 4 Conclusion

Artificial intelligence approaches have been developed with broad applications in biomedical research, including medical imaging processing, diagnostic record processing and antibiotic development. The efforts accumulated in NLP and deep learning domains have led to the development of protein and peptide engineering by deciphering biological cryptography from raw sequences and their related derivative information. Here, we utilized deep learning-based techniques to develop a computational AMP identification method. The method combines a pre-trained transformer architecture and ASL to solve the intrinsic data imbalance within the AMP dataset and improve predictive performance. The functional activities of different pathogen targets and toxicities are important properties for the discovery of novel AMPs. Therefore, we adopted the model for identifying AMPs and predicting their possible targets related to seven different micro-organisms. The evaluation results confirmed the capability of the proposed model to solve data imbalance and improve the encoding representation for AMP sequence prediction. We believe that the proposed scheme combining the transfer-learning-based transformer architecture and imbalanced learning techniques can be widely applied to other biological sequence analysis problems.

## Authors’ contributions

Y.P. and T.-Y.L. conceived the idea and design of this study. Y.P. constructed the implementation of the deep learning model and related analysis pipeline. Y.P. and L.Y. conducted the data pre-processing, model training and evaluation experiments. Y.P. and L.Y. carried out and interpreted the analysis. Z.W. and T.-Y.L. supervised the project.

## Funding

This work was supported by the Guangdong Province Basic and Applied Basic Research Fund (2021A1515012447), National Natural Science Foundation of China (32070659), the Science, Technology and Innovation Commission of Shenzhen Municipality (JCYJ20200109150003938), Ganghong Young Scholar Development Fund (2021E007) and Shenzhen-Hong Kong Cooperation Zone for Technology and Innovation (HZQB-KCZYB-2020056). This work was also supported by the Warshel Institute for Computational Biology, School of Life and Health Sciences, The Chinese University of Hong Kong, Shenzhen, China.

*Conflict of Interest*: none declared.

## Supplementary Material

btac711_Supplementary_Data
