# Supplementary material for: Integrating transformer and imbalanced multi-label learning to identify antimicrobial peptides and their functional activities
Source: Bioinformatics. 2022 Nov 3;38(24):5368–74. doi: 10.1093/bioinformatics/btac711 (PMC9750108; doi:10.1093/bioinformatics/btac711)
Supplement: btac711_Supplementary_Data [file btac711_supplementary_data.zip › btac711_Supplementary_Data/Supplementary.docx]

| **Task** | **Train size** | **Test size** | **Label ratio (%)** |
| --- | --- | --- | --- |
| AMP vs. non-AMP | | | |
| AMP | 3876 | 2584 | 40.58 |
| non-AMP | 9552 | 6369 | - |
| Multi-label target prediction | | | |
| AnGp | 1841 | 789 | 40.71 |
| AnGn | 1832 | 785 | 40.51 |
| AnFu | 1696 | 727 | 37.51 |
| AnVi | 1397 | 599 | 30.90 |
| AnCa | 636 | 273 | 14.07 |
| MamIh | 216 | 93 | 4.78 |
| AnPara | 129 | 55 | 2.85 |

**Table S1** The statistics of the collected dataset for antimicrobial peptides (AMPs) identification and target functional activity prediction, including the number of sequences for train dataset (train size), test dataset (test size) and the ratio of positive instances over the entire dataset for each label. The labels under the second task are anti-Gram-positive bacteria (AnGp), anti-Gram-negative bacteria (AnGn), anti-fungus (AnFu), antivirus (AnVi), anticancer (AnCa), mammalian cell-inhibition (MamIh), and antiparasite (AnPara).

| **Hyperparameter** | **AMP vs. non-AMP** | **Multi-label target prediction** |
| --- | --- | --- |
| Leanrnig rate | 0.04 | |
| Epochs | 240 | 300 |
| Decay time points | [20, 40, 80, 90, 95, 120, 150, 180, 210] | |
| Decay rate | 0.75 | |
| Dropout rate | 0.1 | |
| Focusing parameters for ASL (γ_-_, γ_+_) investigated | {(0, 0), (1, 1), (2, 1), (2, 2), (3, 1), (3, 2), (3, 3), (4, 1), (4, 2), (4, 3), (4, 4), (5, 1), (5, 2), (5, 3), (5, 4), (5, 5)} | |
| Margin of the probability shifting for ASL | 0.1 | |

**Table S2** Preset settlements and hyperparameters of the model establishments in this study. The ASL stands for the asymmetric loss.

| **Sequence** | **dbAMP_ID (Name)** | **UniprotKB ID/AC** | **Source** |
| --- | --- | --- | --- |
| GGLRSLGRKILRAWKKYGPIIVPIIRIG | dbAMP_02744 (Cathelicidin-5) | CTHL5_BOVIN (P54229) | Bovine (Bos taurus) |
| GLPVCGETCFGGTCNTPGCSCTWPICTRD | dbAMP_03732 (Katala-B2) | KAB2_OLDAF (P58454) | Oldenlandia affinis |
| GIGAVLKVLTTGLPALISWIKRKRQQ | dbAMP_02895 (Melittin) | MEL_VESMG (P68408) | Component from honeybee (Apis mellifera) venom. |
| ILPWKWPWWPWRR | dbAMP_04768 (Indolicidin) | CTHL4_BOVIN (P33046) | Bovine (Bos taurus) |
| GRFKRFRKKFKKLFKKLSPVIPLLHLG | dbAMP_03977 (Cathelicidin-6) | CTHL6_BOVIN (P54228) | Bovine (Bos taurus) |

**Table S3** Peptide sequences with covering all functional activities of targets in this study. Related information are collected from dbAMP v2.0.

| **(γ_-_, γ_+_) for ASL** | **AACC (%)** | **SEN (%)** | **SPEC (%)** | **GMean (%)** |
| --- | --- | --- | --- | --- |
| (0,0) | 96.70 | 95.59 | 97.80 | 96.69 |
| (1,1) | 96.73 | 95.82 | 97.64 | 96.73 |
| (2,1) | 96.58 | 95.51 | 97.64 | 96.57 |
| (2,2) | 96.74 | 96.05 | 97.43 | 96.74 |
| (3,1) | 96.85 | 96.28 | 97.43 | 96.85 |
| (3,2) | 96.76 | 95.82 | 97.69 | 96.75 |
| (3,3) | 96.65 | 95.98 | 97.33 | 96.65 |
| (4,1) | 96.70 | 95.78 | 97.61 | 96.69 |
| (4,2) | 96.78 | 95.98 | 97.58 | 96.78 |
| (4,3) | 96.64 | 95.70 | 97.58 | 96.64 |
| (4,4) | 96.80 | 96.09 | 97.50 | 96.79 |
| (5,1) | 96.59 | 95.70 | 97.47 | 96.58 |
| (5,2) | 96.58 | 95.43 | 97.72 | 96.57 |
| (5,3) | 96.66 | 95.59 | 97.74 | 96.66 |
| (5,4) | 96.60 | 95.59 | 97.61 | 96.60 |
| (5,5) | 96.70 | 95.63 | 97.77 | 96.69 |

**Table S4** Comprehensive test performance records of the first task (AMP vs. non-AMP) for the transformer-based asymmetric loss model with different combination of ASL focusing parameters, γ_+_ and γ_-_.

| **Method** | **AACC (%)** | **SEN (%)** | **SPEC (%)** | **GMean (%)** |
| --- | --- | --- | --- | --- |
| First task (AMP v.s. non-AMP) | | | | |
| Ours | 96.85 | 96.28 | 97.43 | 96.85 |
| AMPScanner v2 | 67.75 | 71.59 | 63.91 | 67.64 |
| iAMPpred | 47.28 | 80.41 | 14.16 | 33.74 |
| ClassAMP | 48.91 | 97.93 | 0.63 | 2.49 |
| AnGp | | | | |
| Ours | 85.78 | 87.96 | 83.60 | 85.75 |
| iAMPpred | 65.36 | 89.91 | 40.81 | 60.58 |
| ClassAMP | 49.81 | 30.65 | 68.98 | 45.98 |
| AnGn | | | | |
| Ours | 85.90 | 87.26 | 84.53 | 85.89 |
| iAMPpred | 65.21 | 89.83 | 40.60 | 60.39 |
| ClassAMP | 49.47 | 30.24 | 68.70 | 45.58 |
| AnFu | | | | |
| Ours | 79.24 | 86.66 | 71.82 | 78.89 |
| iAMPpred | 69.62 | 90.80 | 48.44 | 66.32 |
| ClassAMP | 46.08 | 38.16 | 54.00 | 45.39 |
| AnVi | | | | |
| Ours | 86.63 | 88.81 | 84.44 | 86.60 |
| iAMPpred | 34.72 | 46.49 | 22.95 | 32.66 |
| ClassAMP | 48.65 | 24.24 | 73.06 | 42.09 |
| AnCa | | | | |
| Ours | 78.56 | 70.33 | 86.79 | 78.13 |
| AntiCP | 52.94 | 12.82 | 93.07 | 34.54 |

**Table S5** Comparative performance analysis for this study (Ours) with other available AMP prediction tools. The focusing parameters of asymmetric loss are (γ_-_ = 3, γ_+_ = 1) for the first task and (γ_-_ = 5, γ_+_ = 1) for the second task.

| **Target** | **Method** | **AACC (%)** | **SEN (%)** | **SPEC (%)** | **GMean (%)** |
| --- | --- | --- | --- | --- | --- |
| AnGp | Baseline | 83.11 | 74.33 | **91.89** | 82.64 |
|  | Transformer + CE | 85.19 | 82.89 | 87.48 | 85.15 |
|  | Transformer + ASL | **85.78** | **87.96** | 83.60 | **85.75** |
| AnGn | Baseline | 82.88 | 74.26 | **91.49** | 82.43 |
|  | Transformer + CE | 85.64 | 83.06 | 88.22 | 85.6 |
|  | Transformer + ASL | **85.90** | **87.26** | 84.53 | **85.89** |
| AnFu | Baseline | **80.22** | 68.52 | **91.91** | 79.36 |
|  | Transformer + CE | 80.17 | 74.55 | 85.79 | **79.97** |
|  | Transformer + ASL | 79.24 | **86.66** | 71.82 | 78.89 |
| AnVi | Baseline | 85.18 | 75.25 | **95.10** | 84.6 |
|  | Transformer + CE | 85.48 | 80.47 | 90.48 | 85.33 |
|  | Transformer + ASL | **86.63** | **88.81** | 84.44 | **86.60** |
| AnCa | Baseline | 66.18 | 34.80 | **97.55** | 58.26 |
|  | Transformer + CE | 68.89 | 43.59 | 94.18 | 64.07 |
|  | Transformer + ASL | **78.56** | **70.33** | 86.79 | **78.13** |
| MamIh | Baseline | 56.31 | 12.90 | **99.72** | 35.87 |
|  | Transformer + CE | 59.51 | 20.43 | 98.58 | 44.88 |
|  | Transformer + ASL | **78.83** | **65.59** | 92.08 | **77.71** |
| AnPara | Baseline | 54.52 | 9.09 | **99.95** | 30.14 |
|  | Transformer + CE | 56.05 | 12.73 | 99.36 | 35.56 |
|  | Transformer + ASL | **63.88** | **30.91** | 96.84 | **54.71** |

**Table S6** Performance categorized by each functional activity target on the multi-label classification of the second task. CE stands for the standard cross entropy (γ_−_ = 0, γ_+_ = 0). The focusing parameters of ASL here are (γ_−_ = 5, γ_+_ = 1).

| **(γ_-_, γ_+_) for ASL** | **AACC (%)** | **SEN (%)** | **SPEC (%)** | **GMean (%)** |
| --- | --- | --- | --- | --- |
| (0,0) | 74.41% | 56.82% | 92.01% | 68.65% |
| (1,1) | 74.93% | 57.70% | 92.16% | 69.55% |
| (2,1) | 76.91% | 62.90% | 90.92% | 73.23% |
| (2,2) | 75.87% | 59.61% | 92.13% | 71.16% |
| (3,1) | 77.48% | 65.91% | 89.05% | 74.19% |
| (3,2) | 76.72% | 62.88% | 90.56% | 72.89% |
| (3,3) | 76.36% | 61.46% | 91.26% | 72.29% |
| (4,1) | 78.23% | 68.94% | 87.52% | 75.65% |
| (4,2) | 77.95% | 67.75% | 88.16% | 75.03% |
| (4,3) | 77.39% | 64.62% | 90.17% | 74.17% |
| (4,4) | 76.06% | 60.41% | 91.72% | 71.74% |
| (5,1) | 79.83% | 73.93% | 85.73% | 78.24% |
| (5,2) | 78.46% | 69.90% | 87.02% | 75.73% |
| (5,3) | 78.38% | 69.02% | 87.74% | 75.71% |
| (5,4) | 77.57% | 65.71% | 89.43% | 74.27% |
| (5,5) | 76.04% | 60.65% | 91.43% | 71.59% |

**Table S7** Comprehensive test performance metrics averaged across all the corresponding metrics of target labels under the second task for the transformer-based asymmetric loss model with different combination of ASL focusing parameters, γ_+_ and γ_-_.


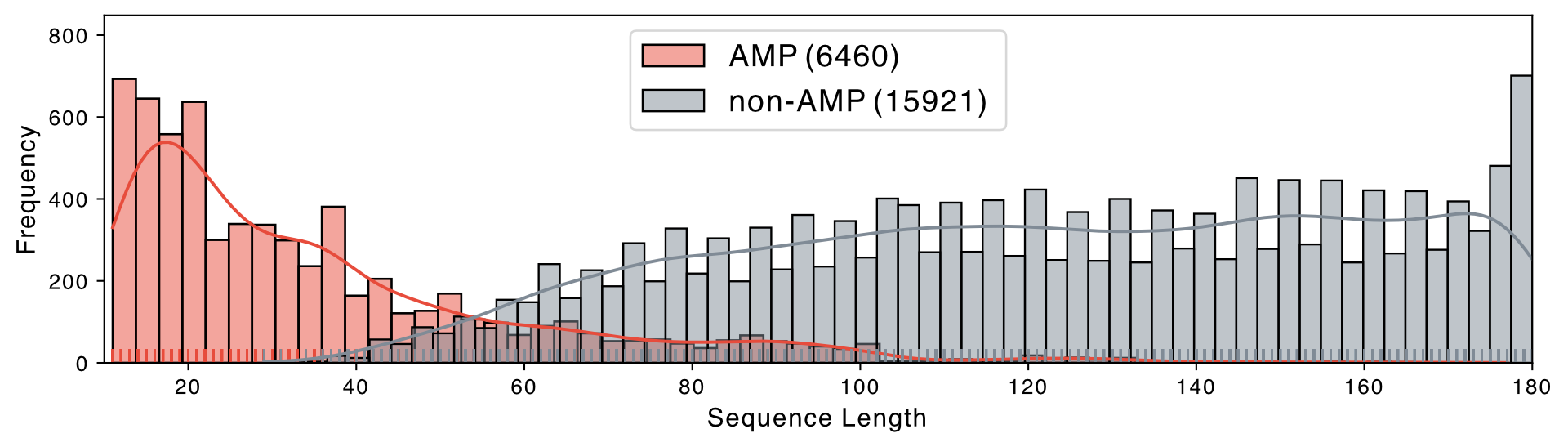


**Figure S1** The length distributions of AMPs and non-AMPs.

The peptide descriptor mentioned in the article is defined as follows:

1. Amino acid composition (ACC)

The amino acid composition of a protein is one of the critical features, which is defined as the frequency of an amino acid in a sequence and it can be calculated as follows:


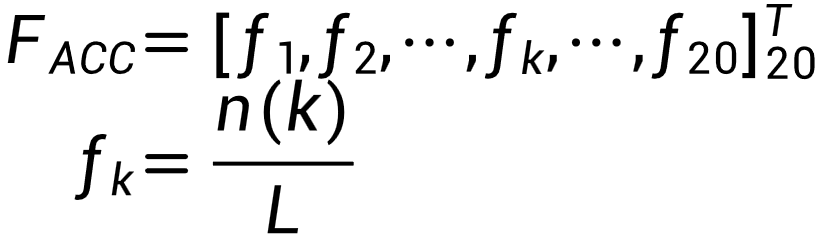


where ***L*** denotes the length of the peptide sequence, and ***n(k)*** represents the occurrence of amino acid. From the definition of ACC, it is a 20-dimensional vector, and the sum of all dimensions is ***1.***

2. Dipeptide composition (DPC)

Similar to the amino acid composition, DPC calculates the frequency of occurrence of an amino acid pair, which can be calculated as follow:


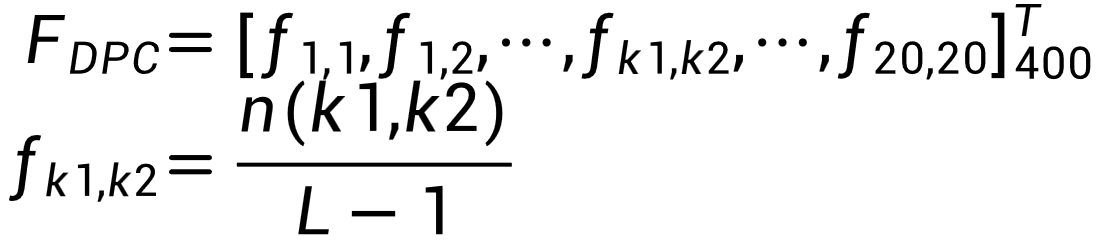


where ***n(k1,k2)*** represent the frequency of occurrence of an amino acid pair ***(k1,k2)***. Since there are a total of 20 amino acids that make up proteins, ***(k1,k2)*** has a total of ***20 × 20 = 400*** possible combinations. Therefore, DPC is a 400-dimensional vector, and the sum of all dimensions is equal to one.

3.Composition of k-spaced amino acid group pairs (CKSAAGP)

This approach is based on a deeper understanding of the biological significance of amino acids. 20 amino acids can be divided into five classes according to their physicochemical properties (**Table S8**): aliphatic, aromatic, positive-charged, negative-charged and uncharged residues. Assuming that two amino acids belong to the aliphatic and negatively-charged amino acids and 'X' represents any residues, 'aliphatic.X.X.negatively-charged' is an example of a 2-spaced amino acid group pair. Next, CKSAAGP calculates the frequency of occurrence of a particular amino acid pair separated by ***k*** arbitrary residues.

| **Category** | **Amino acids** |
| --- | --- |
| Aliphatic | G, A, V, L, M, I |
| Aromatic | F, Y, W |
| Positive-charged | K, R, H |
| Negative-charged | D, E |
| Uncharged | S, T, C, P, N, Q |

**Table S8** The categories of amino acids grouped by physiochemical properties

4.Pseudo amino acid composition (PAAC)

PAAC is another valid peptide descriptor representing amino acid sequences and has been used in many bioinformatics analyses. AAC, DPC and CKSAAGP represent the amino acid composition of the peptide but ignore sequence-order information. PAAC dealt with sequence order effects by using a set of discrete factors, which can be represented as follow:


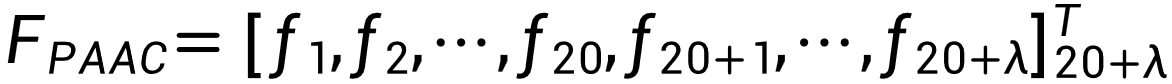


The discrete counted-rank correlation ***λ*** is an essential parameter for PAAC that determines the feature dimension. Another parameter for PAAC is the weighting factor ***ω***, which determines the importance of the sequence order in the PAAC descriptor. Specifically, the larger ***ω*** is, the more the sequence order is considered.

5. Physicochemical features (PHYC)

In general, physicochemical properties of peptides and their functional activity are closely related. Here, we chose eight representative physicochemical properties as our descriptors, including transmembrane propensity, hydrophobicity, net charge, isoelectric point, alpha helical propensity, aliphatic index, Boman index and hydrophobic moment.
